# Clinical Impact of Single Nucleotide Polymorphism in PD-L1 on Response to Nivolumab for Advanced Non-Small-Cell Lung Cancer Patients

**DOI:** 10.1038/srep45124

**Published:** 2017-03-23

**Authors:** Takashi Nomizo, Hiroaki Ozasa, Takahiro Tsuji, Tomoko Funazo, Yuto Yasuda, Hironori Yoshida, Yoshitaka Yagi, Yuichi Sakamori, Hiroki Nagai, Toyohiro Hirai, Young Hak Kim

**Affiliations:** 1 Department of Respiratory Medicine, Graduate School of Medicine, Kyoto University, 54 Kawahara-cho, Shogoin, Sakyo-ku, Kyoto 606-8507, Japan.

## Abstract

This study was intended to determine the efficacy of nivolumab, we evaluated treatment response with respect to PD-1/PD-L1 SNPs among patients with NSCLC. A total of 50 patients with NSCLC were treated with nivolumab and were also evaluated for PD-1/PD-L1 single nucleotide polymorphisms (SNPs) from plasma DNA. We investigated the association among PD-1/PD-L1 SNPs, objective response rate (ORR) and progression-free survival (PFS). Two of seven SNPs studied showed association with ORR and PFS, with maximum evidence at the marker rs2282055. The ORR was 25%, 15%, and 0% for the G/G, G/T and T/T genotypes of PD-L1 rs2282055, respectively. The G allele of PD-L1 rs2282055 was significantly associated with better clinical response compared with the T allele (P = 0.0339 [Cochran-Armitage trend test]). The median PFS time was 2.6 months (95% confidence interval [CI], 1.8 months to 4.3 months) for the G/G and G/T genotypes and 1.8 months (95% confidence interval [CI], 0.4 months to 2.2 months) for the T/T genotype (P = 0.0163). Moreover, the C/C and C/G genotypes of PD-L1 rs4143815 were significantly associated with better ORR and PFS in NSCLC patients treated with nivolumab. These results suggest that rs2282055 and rs4143815 may be a biomarker for the efficacy of nivolumab.

Although lung cancer remains a major cause of cancer-related mortality worldwide, targeted therapies and immunotherapy contribute to better clinical outcome in non–small-cell lung cancer (NSCLC) patients^1^. Nivolumab, a fully human IgG4 programmed death 1 (PD-1) immune checkpoint inhibitor antibody, targets the PD-L1 protein and has demonstrated improved patient survival over docetaxel in previously treated advanced NSCLC^2^,^3^. It was approved by the U.S. Food and Drug Administration for treating lung cancer in October 2015 and approved for second-line treatment after platinum-based first-line chemotherapy. The PD-1 receptor expressed on activated T-cells interacts with the tumor-expressing ligands PD-L1 and PD-L2 to down-regulate the T-cell-promoted tumor immune escape mechanism^2^,^3^,^4^. Nivolumab binds to PD-1 and inhibits the binding of the receptor to its ligands PD-L1 and PD-L2 on tumor cells, and inhibits the co-inhibitory signals on T-cells^5^. This leads to the attenuation of T-cell suppression and induces antitumor responses.

Some patients with lung cancer show marked responses to nivolumab, whereas others do not respond (ORR ~20%). It is currently difficult to predict treatment response to nivolumab since there are no precise biomarkers for it. Expression of PD-L1 in the tumor microenvironment is thought to be crucial for therapeutic activity^6^; however, further investigations suggest that the expression of PD-L1 is not an ideal biomarker since the PD-L1 expression assessed by immunohistochemistry and the associated response to immune checkpoint inhibitors are not always concordant^7^,^8^. The expression of PD-L1 may not predict therapeutic efficacy because the interactions between PD-1 and PD-L1 on B-cells link the adaptive and innate immune systems, which may explain the changes in PD-1/PD-L1 response to cancer during cancer treatment^9^. Furthermore, it is difficult to compare PD-L1 expression levels among various studies since different immunohistochemistry detection antibodies for PD-L1 staining are used and specific PD-L1 expression cut-off levels vary among these studies^10^. T-cell receptor clonality^11^, co-expression of other immunologic markers^12^, somatic mutational burden^13^, and PD-L1-expressing circulating tumor cells^14^ are also candidate biomarkers for the PD-L1 blockade therapy; however, these alternatives are lacking in conclusive evidence.

Recently, the application of next-generation sequencing (NGS) has emphasized the potential role of somatic DNA sequence markers, which may be used as independent markers, novel therapeutic targets, or as tools to refine expression markers^15^. Similarly, progress in specific and genome-wide single nucleotide polymorphisms (SNP) arrays have underlined the role of germline mutations in modifying cancer and treatment outcome^16^. Several studies have indicated that genetic polymorphism was correlated with clinical outcome in NSCLC^17^. Moreover, some studies have reported monochronal antibody therapy to be related with polymorphisms of the target molecule’s gene, for example, CTLA4 polymorphism with antiCTLA4^18^,^19^, CD52 with alemtuzmab^20^, Il-6 or C1qA with rituximab^21^,^22^, and Fc gamma receptor 3a with ofatumumab^23^. Therefore, we hypothesized that germline PD-1/PD-L1 SNPs might be a predictive biomarker. Some SNPs of PD-1/PD-L1 were previously shown to have clinical importance to diseases such as Addison’s disease^24^, gastric adenocarcinoma^25^, NSCLC^26^, and type 1 diabetes^27^. Furthermore, a recently reported study suggested the association between a PD-L1 single nucleotide polymorphism and the efficacy of first-line paclitaxel-cisplatin chemotherapy^28^. In this study, we hypothesized that polymorphisms of the PD-L1 gene may alter the immune checkpoint function, thereby influencing clinical outcomes of response to immune checkpoint inhibitors in patients with lung cancer. We analyzed the association between polymorphism in the PD-L1 and PD-1 genes and clinical response to nivolumab in NSCLC patients.

## Results

### Patient Characteristics and Clinical Outcome

Patient characteristics are listed in Table 1. The median age was 65 years (40 to 80 years), and 34 patients (68%) were men. Fifty patients (100%) had an Eastern Cooperative Oncology Group (ECOG) performance status of 0 or 1. Forty patients (80%) were histologically diagnosed with adenocarcinoma and 10 patients were diagnosed with squamous cell carcinoma. Nivolumab was administered to all patients; 10 patients (20%) received the drug as the second-line treatment and 40 patients (80%) received it as the third-line treatment or beyond.

Among the 50 patients, 8 (16%) had PR, 16 (32%) had stable disease (SD), 26 (52%) had PD (Table 1), and none had obtained complete response. Objective responses were observed in 8 (16%) patients. The ORR for this study was similar to those observed in recent large clinical trials of nivolumab in NSCLC^2^,^3^. The median PFS time in all patients was 2.2 months (95% confidence interval [CI], 1.8 to 3.3 months). The ORR was not significantly associated with age, gender, smoking status, histology or treatment line of nivolumab.

### Associations Between Genotypes and Clinical Outcome

Five PD-L1 SNPs and two PD-1 SNPs were genotyped in 50 NSCLC patients who were administered with nivolumab. The SNP genotype, gene information, genotype frequency, and the associated clinical response are shown in Table 2. The G allele of PD-L1 rs2282055 and the C allele of PD-L1 rs4143815 were significantly associated with better clinical response compared to the T allele of rs2282055 (P = 0.0339, Cochran-Armitage trend test) and to the G allele of rs4143815 (P = 0.0319, Cochran-Armitage trend test), respectively. No significant association between genotypes and ORR was observed for the remaining SNPs.

### Association of PD-L1 rs4143815 with PFS

**Figure 1:**
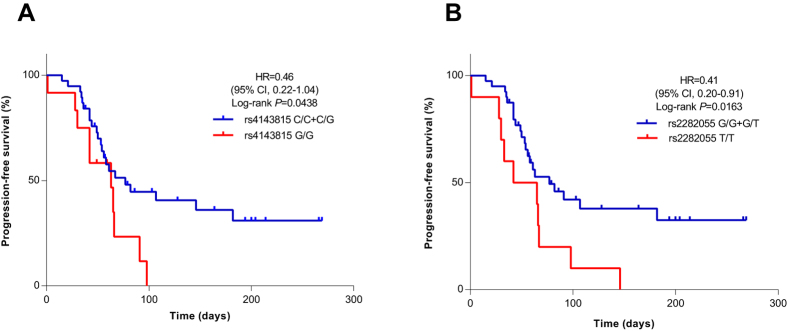
Kaplan-Meier curves of PFS after administration of nivolumab, stratified by the genotype PD-L1 rs4143815 (**A**) and rs2282055 (**B**). Symbols denote censored observations. The differences between the groups were evaluated using the log-rank test. Multivariate Cox proportional hazards models were used to estimate adjusted hazard ratios (HR) with 95% confidence intervals.

**Figure 2:**
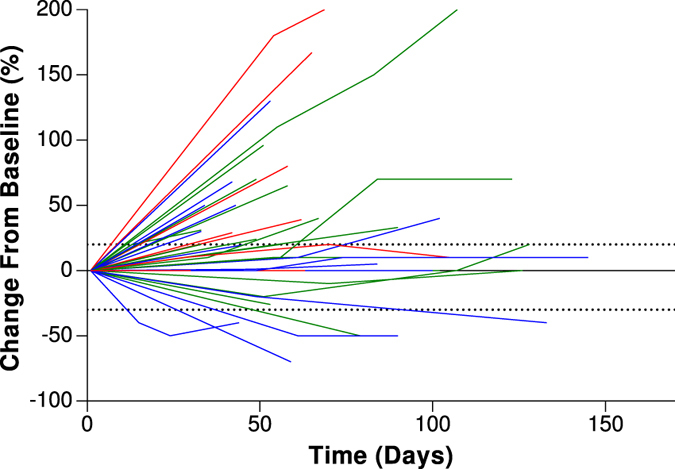
Best overall responses in all patients in this cohort with nivolumab. The blue lines represent the C/C genotype, the green lines represent the C/G genotype, and the red lines represent the G/G genotype of PD-L1 rs4143815. Duration of best overall responses and changes in target lesions from baseline in 50 patients who received nivolumab.

Of the 50 patients treated with nivolumab, 12 were G/G (24%), 15 were C/C (30%), and 23 were C/G (46%) of PD-L1 rs4143815. Genotype was not associated with clinicopathologic variables (Table 3). The C/C genotype was more associated with treatment effect compared to both C/G and G/G genotypes. Objective responses were observed in 4 of 15 (27%), 4 of 23 (17%), and none of 12 (0%) patients of PD-L1 rs4143815 genotypes C/C, C/G and G/G, respectively. Kaplan-Meier survival curves (Fig. 1A) also show associations of PFS among patients with the C/C, C/G, and G/G genotypes. The median PFS time was 2.6 months (95% confidence interval [CI], 1.8 months to 6.1 months) for the C/C and C/G genotypes and 2.1 months (95% confidence interval [CI], 0.9 months to 3.0 months) for the G/G genotype (P = 0.0438). As shown in Fig. 2, patients with the C/C and C/G genotypes of PD-L1 rs4143815 showed slightly better responses to nivolumab.

### Association of PD-L1 rs2282055 with PFS

We also assessed PFS among the PD-L1 rs2282055 genotypes. Objective responses were observed in 5 of 20 (25%), 3 of 20 (15%), and none of 10 patients of PD-L1 rs2282055 genotypes G/G, G/T and T/T, respectively (Table 3). Kaplan-Meier curves showed significant differences among the G/G, G/T, and T/T genotypes (Fig. 1B). The median PFS time was 2.6 months (95% confidence interval [CI], 1.8 months to 4.3 months) for the G/G and G/T genotypes and 1.8 months (95% confidence interval [CI], 0.4 months to 2.2 months) for the T/T genotype (P = 0.0163).

### Haplotype analysis

Three polymorphism of the PD-L1 gene, rs822339, rs2282055, and rs1411262, were generally under strong linkage disequilibrium (LD) from the pairwise disequilibrium map (LD map) (Supplementary Fig. S1). The two SNPs, rs2282055 and rs4143815, were in LD (|D’| = 0.624 and r^2^ = 0.292) in this study. In the PD-1 gene, the D’ value and r^2^ between rs2227981 and rs2227982 were |D’| = 1.0 and r^2^ = 0.355, respectively.

## Discussion

We assessed the efficacy of nivolumab according to PD-1/PD-L1 SNPs. Interestingly, our study showed that patients with the C/C or C/G genotype of PD-L1 rs4143815 and the G/G or G/T genotypes of rs2282055 had a significantly longer PFS with nivolumab treatment than patients with the G/G genotype and T/T genotype respectively. In addition, none of the patients obtained treatment effect with the G/G genotype of PD-L1 rs4143815 and the T/T genotype of rs2282055.

Currently, since the known candidate biomarkers of nivolumab mainly originate from the tumor itself and the surrounding tumor environment, it was suggested that the PD-L1 expression in tumor cells measured by immunohistochemistry may predict responses to immune checkpoint inhibitors^6^,^29^. A meta-analysis indicated that the benefit from PD-1 inhibitors versus docetaxel in second-line treatment of NSCLC is limited to the PD-L1 > 1% subpopulation^30^. However, tumors that do not express detectable levels of PD-L1 on the cell surface can also respond to immune checkpoint inhibitors. A recent study reported that PD-L1 expression changes during the course of clinical treatment^31^, which suggests that the PD-L1 expression measured by immunohistochemistry cannot be solely used as the predictive treatment biomarker. A better biomarker for predicting response to nivolumab is required to help clinical decision-making and potentially help expose fewer patients to inadequate treatments and their associated toxicities and costs.

PD-L1 rs4143815, which is located in the 3′ -untranslated region (UTR) and binds to miR-570^25^,^32^, has been suggested by several studies to have medical significance. The C/C genotype of PD-L1 rs4143815 is associated with increased risk of gastric cancer due to its interference with the miR-570 function and by possibly suppressing the immunological tumor restriction through increasing PD-L1 expression^25^. S. Lee and colleagues reported that the C/C genotype of PD-L1 rs4145815 is associated with worse clinical outcomes for patients who have been administered first-line paclitaxel-cisplatin chemotherapy^28^ due to the unstable binding of miRNAs to PD-L1 mRNA, which is caused by the increased minimum free energy produced from the RNAhybrid. The G/G genotype of PD-L1 rs4143815 is associated with type 1 diabetes and has also been observed with lower serum levels of the PD-L1 protein in patients^27^. In addition, X. Shi and colleagues have recently reported that patients who underwent liver transplantation and received a donor liver carrying the C allele of PD-L1 rs4143815 had a decreased risk in developing late acute rejection^33^. They also found that hepatic BDCA1-positive dendritic cells with the rs4143815 C/C genotype showed higher PD-L1 expression than those with the G/G genotype after IFN-gamma stimulation^33^. These studies suggest that the C allele of PD-L1 rs4143815 increases PD-L1 expression by attenuating the miR-570 function. These findings could explain the reason why patients with the G/G genotype of PD-L1 rs4143815 did not respond to nivolumab in our study. Therefore, the genotypes of PD-L1 rs4143815 may predict the level of expression of PD-L1 on the cell surface throughout the course of treatment. Moreover, a recent study suggested that the disruption of the PD-L1 3′-UTR, which is caused by the structural variation of the gene transformation of the PD-L1 3′-UTR region, increases PD-L1 expression and leads to immune escape of tumor cells^34^. The mechanism for immune escape, which occurs during tumor growth^35^, involves the avoidance of first tumor antigen recognition by B-cells and unresponsive general cytotoxic T lymphocytes (CTL). By increasing PD-L1 expression levels and thereby down-regulating T-cell response, the C/C genotype of PD-L1 rs4143815 may be involved in the escape from immunosurveillance. Previous report showed that Grave disease and Addison’s disease related to rs2282055, but the function of PD-L1 rs2282055 is unknown^24^. The two SNPs, rs2282055 and rs4143815, were in relatively weak LD (r^2^ < 0.8). This might explain the clinical significance of PD-L1 rs2282055. Conversely, PD-L1 rs822339, PD-L1 rs1411262, and PD-L1 rs2282055 were in strong LD (Supplementary Fig. S1), therefore it is possible that these haplotypes might affect the efficacy of nivolumab.

In this study, we focused on the PD-1/PD-L1 SNPs as a predictive biomarker of nivolumab since the PD-1/PD-L1 pathway is involved in the immune system. The PD-1 receptor, which is broadly expressed in multiple cell types, including T- and B-cells, dendritic cells, and macrophages^36^, plays a role in down-regulating T-cell responses^37^,^38^ and leads to immune suppression instead of activation. So far, the function of the PD-L1 polymorphism has not been fully investigated; however, it may modify anti-tumor immunity and affect the response to chemotherapy or immune checkpoint inhibitors.

This study had several limitations. First, this was a retrospective study with a small sample size and the patients who had received nivolumab were already relatively heavily pre-treated. Nevertheless, we showed a clinically meaningful difference in response rates and PFS among the PD-L1 rs4143815 and rs2282055 genotypes. Second, since this study lacks in immunohistochemical information from biopsies, the association between the PD-L1 rs4143815 genotypes and the PD-L1 expression in tumor cells is unknown in this cohort. Although PD-L1 expression is an important biomarker, we did not assess its expression in this study. The reason for this is due to the use of different antibodies in immunohistochemistry assays and the varying cut-off values to evaluate the expression of PD-L1, which make it difficult to compare with other studies. Third, we selected seven PD-1/PD-L1 polymorphisms that are thought to be functional based on previous reports; however, there may be other, more efficient predictive SNP biomarkers.

The association between PD-L1 SNPs and the efficacy of nivolumab in a large-sized study needs to be investigated in the future. We are planning future studies to assess the association of PD-L1 expression, PD-L1 SNPs and efficacy of anti-cancer treatment in order to confirm the observations presented in this study.

In conclusion, we found that in advanced stage NSCLC patients who received nivolumab, the C allele of PD-L1 rs4143815 and the G allele of rs2282055 were significantly associated with better ORR and PFS. This is the first report that PD-L1 SNP, which was thought to increase PD-L1 expression, is associated with a response to nivolumab. Therefore, these results suggests that PD-L1 rs4143815 and rs2282055 may be a biomarker for identifying patients for whom nivolumab may be particularly beneficial, which, as a result, may contribute to the clinical setting in the treatment of lung cancer.

## Patients and Methods

### Patients

Between December 2015 and August 2016, a total of 67 consecutive patients with histologically or cytologically confirmed NSCLC were treated with nivolumab. Among these 67 patients, we enrolled 50 patients in this study, all of whom were registered at Kyoto University Hospital. The remaining 17 patients were excluded from this study as a result of lack of DNA samples, discontinued observation due to change in hospital, the inability to obtain informed consent, or the patient had a history of double cancer. Participants in this study have provided a written informed consent and all experimental methods were in accordance with the 1975 Declaration of Helsinki. The study was approved by the Review Board of Kyoto University Hospital. A 10-mL peripheral blood sample was collected from each study participant. This procedure was performed in accordance with the ethical principles for medical research.

### Genotyping and SNP selection

Genomic DNA was extracted from peripheral blood leukocytes using the GENE PREP STAR NA-480 (KURABO, Osaka, Japan) according to the manufacturer’s instructions. Genotyping was performed using the TaqMan^®^ genotyping assay (Applied Biosystems, Foster City, CA) and analyzed with an Applied Biosystems 7300 Real-Time PCR System (Applied Biosystems). The polymerase chain reaction (PCR) solution included 12.5 μL of 2X TaqMan Universal PCR Master Mix, 0.3125 μL of primer probe mix, 11.2 μL of nuclease-free water, and 1 μL of DNA sample. After baseline fluorescence measurements at 25 °C, the PCR protocol included a 10-min incubation at 95 °C, 40 cycles of denaturing at 92 °C for 15 s and annealing and extending at 60 °C for 1 min, and a final measurement of fluorescence at 60 °C. SNPs with previously described associations or putative functional effects were selected^24^,^25^,^26^,^27^,^28^,^39^,^40^,^41^,^42^,^43^.

### Evaluation of Nivolumab Efficacy

Medical records were reviewed and data extracted based on clinical features and treatment histories. These data have been updated as of October 2016. The initial dose of nivolumab was 3 mg/kg every two weeks and was intravenously administered until the assessment of progressive disease (PD) or an unacceptable toxicity level was reached. Radiographic imaging was done every 4 to 6 weeks. Response was assessed as per the Response Evaluation Criteria in Solid Tumors (RECIST) (version 1.1) by an investigator. The PFS was measured from the start of nivolumab administration until the date of RECIST PD. Patients without documented clinical or radiographic disease progression were censored on the date of the last follow-up. The ORR, as determined by the RECIST criteria, was calculated as the total percentage of patients with a complete or partial response (PR).

### Statistical analysis

The JMP Pro statistical software version 12.1.0 (SAS Institute Inc, Tokyo, Japan) was used for all statistical analyses. Differences in characteristics among the genotypes were compared using Fisher’s exact test for categorical data. Genotypes were analyzed separately and as combined allelic groups. Clinical responses among the genotypes were analyzed using the Cochrane-Armitage test. The patient’s baseline characteristics and the genotype of rs4143815 or rs2282055 were analyzed using one-way analysis of variance (ANOVA) for continuous variables.

The survival curves were estimated using the Kaplan-Meier method. Statistical significance of the relationship between PFS and genetic polymorphism was assessed using the trend log-rank-test. Multivariate Cox proportional hazards models were used to estimate adjusted hazard ratios (HR) with 95% confidence intervals. Multivariable regressions analysis was adjusted for age, gender, and histology. Haploview v4.2 was used to calculate the linkage disequilibrium (LD), expressed as D’ and r^2^.

## Additional Information

**How to cite this article:** Nomizo, T. *et al*. Clinical Impact of Single Nucleotide Polymorphism in PD-L1 on Response to Nivolumab for Advanced Non-Small-Cell Lung Cancer Patients. *Sci. Rep.* **7**, 45124; doi: 10.1038/srep45124 (2017).

**Publisher's note:** Springer Nature remains neutral with regard to jurisdictional claims in published maps and institutional affiliations.

## Supplementary Material

Supplementary Information

## Figures and Tables

**Table 1:** Patient Characteristics.

Characteristics	No. of Patients (N = 50) (%)	No. of Responses	ORR (%)	P
Total Patients	50	8	16.0	
Partial Response (PR)	8			
Stable Disease (SD)	16			
Progression of Disease (PD)	26			
Median Age, (Range)	65 (40–80)			
Sex				1.000*
Male	34	7	20.6	
Female	16	1	6.3	
PS				
0–1	50			
2–3	0			
Smoking Status				0.423*
Never	15	1	6.7	
Ever	35	7	20.0	
Histology				0.665*
Adenocarcinoma	40	6	15.0	
EGFR-mutated	(13	2	15.4)	
Squamous Cell Carcinoma	10	2	20.0	
Stage				
I or II	0			
IIIB/IV	50			
Treatment Line				0.665*
First	0			
Second	10	2	20.0	
≥Third	40	6	15.0	

Abbreviations: PS, performance status.

*P values are estimated by Fisher’s exact test.

**Table 2:** Association of PD-L1 and PD-1 Genotypes with Clinical Response.

	Gene	Location	Ancestral allele	No. of Patients	Clinical Response (ORR)		
					No.	%	P†
rs1411262	PD-L1	intron	T				0.0751
T/T				15	4	24	
T/C				21	3	14	
C/C				14	1	7	
rs2282055	PD-L1	intron	T				*0.0339*
T/T				10	0	0	
T/G				20	3	15	
G/G				20	5	25	
rs4143815	PD-L1	3′ UTR	G				*0.0319*
G/G				12	0	0	
G/C				23	4	17	
C/C				15	4	27	
rs2890658	PD-L1	intron	C				0.2088
C/C				33	6	18	
C/A				14	2	14	
A/A				3	0	0	
rs822339	PD-L1	intron	G				0.0855
G/G				14	1	7	
G/A				21	3	14	
A/A				15	4	27	
rs2227981	PD-1	synonymous	G				0.1428
G/G				26	3	12	
G/A				21	4	19	
A/A				3	1	33	
rs2227982	PD-1	missense	G				0.3628
G/G				15	4	27	
G/A				22	1	5	
A/A				13	3	23	

Abbreviations: ORR, objective response rate; ^†^Exact test of Cochran-Armitage trend test across genotypes.

**Table 3:** Clinical Characteristics According to PD-L1 rs4143815 and rs2282055.

Characteristics	rs4143815				rs2282055			
	No. of Patients (N = 50) (%)				No. of Patients (N = 50) (%)			
	C/C	C/G	G/G	P*	A/A	A/C	C/C	P*
Median Age (Range)	64.8 (42–78)	64.1 (40–80)	67.3 (54–77)	n.s.	68.4 (54–74)	63.6 (40–78)	64.9 (41–80)	n.s.
Sex								
Male	11	17	6	n.s.	6	16	12	n.s.
Female	4	6	6	n.s.	4	4	8	n.s.
PS								
0–1	15	23	12	n.s.	10	20	20	n.s.
2–3	0	0	0		0	0	0	
Histology								
Adeno	13	15	12	n.s.	8	16	13	n.s.
EGFR-mutated	6	4	3	n.s.	3	4	6	n.s.
Squamous	2	8	0	n.s.	2	3	5	n.s.
Stage								
I or II	0	0	0		0	0	0	
IIIB/IV	15	23	12	n.s.	10	20	20	n.s.
Treatment Line								
First	0	0	0		0	0	0	
Second	5	2	3	n.s.	2	1	7	n.s.
≥Third	10	21	8	n.s.	8	19	13	n.s.
Treatment Response								
PR	4	4	0		0	3	5	
SD	3	9	4		4	5	7	
PD	8	10	8		6	12	8	
ORR, %	36	17	0	*0.0319*	0	15	25	*0.0339*
DCR, %	47	57	33	n.s.	40	40	60	n.s.

Abbreviations: PS, performance status; PR, partial response; SD, stable disease; PD, progressive disease; ORR, objective response rate; DCR, disease control rate; n.s, not significant.

P values indicate whether the group is equally distributed across the subcategories using analysis of variance, and Cochrane-Armitage test, where appropriate.
