# Supplementary material for: Clinical Impact of Single Nucleotide Polymorphism in PD-L1 on Response to Nivolumab for Advanced Non-Small-Cell Lung Cancer Patients
Source: Sci Rep. 2017 Mar 23;7:45124. doi: 10.1038/srep45124 (PMC5362817; doi:10.1038/srep45124)
Supplement: Supplementary Information [file srep45124-s1.pdf]

Clinical Impact of Single Nucleotide Polymorphism in PD-L1 on Response to Nivolumab for Advanced Non-Small-Cell Lung Cancer Patients

Takashi Nomizo, Hiroaki Ozasa*, Takahiro Tsuji, Tomoko Funazo, Yuto Yasuda, Hironori Yoshida, Yoshitaka Yagi, Yuichi Sakamori, Hiroki Nagai, Toyohiro Hirai, Young Hak Kim,

Department of Respiratory Medicine, Graduate School of Medicine, Kyoto University

Corresponding author: ozahiro@kuhp.kyoto-u.ac.jp

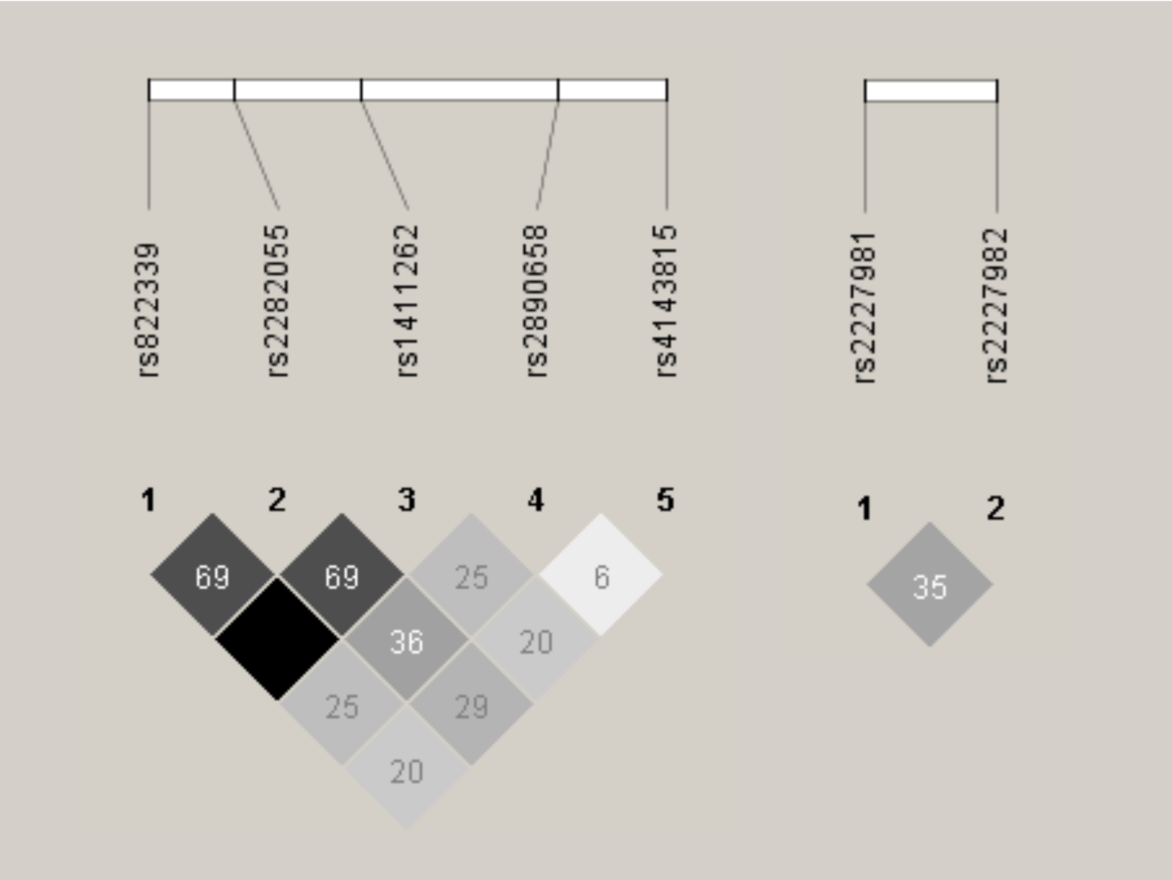

Supplementary Fig.S1.

Linkage disequilibrium (LD) pattern of the PD-L1 and PD-1 SNPs in this study population.

Each number in the squares indicates the r2 index of the LD between the correspondent SNPs.
